# Quince Powder Increases Antioxidant Activity and Probiotic Survival in Yoghurt

**DOI:** 10.17113/ftb.62.04.24.8417

**Published:** 2024-12

**Authors:** Şenay Burak Çınar, Gülfem Ünal, Gülşah Çalışkan Koç, Safiye Nur Dirim, Ayşe Sibel Akalın

**Affiliations:** 1 Republic of Turkey Karabağlar District Directorate of Agriculture and Forestry, Izmir, Turkey; 2 Department of Dairy Technology, Faculty of Agriculture, Ege University, Bornova, Izmir, Turkey; 3 Eşme Vocational School, Food Processing Department, Food Technology Program, Uşak University, Eşme, Uşak, Turkey; 4 Department of Food Engineering, Faculty of Engineering, Ege University, Bornova, Izmir, Turkey

**Keywords:** quince antioxidants, *Bifidobacterium lactis* BB-12®, quince powder, yoghurt

## Abstract

**Research background:**

In recent years, there has been a growing interest in incorporating fruit-based additives into yogurt formulations as a means to improve the functionality of the product. This study aims to produce a functional product by incorporating quince, which is rich in fibre, vitamins, minerals and antioxidant activity, into a yoghurt formulation.

**Experimental approach:**

The influence of the addition of quince powder (0 (control), 0.5, 1.0 and 1.5 %) on the antioxidant and proteolytic activities and culture viability of probiotic yoghurt was investigated for 28 days.

**Results and conclusions:**

The viable counts of yoghurt bacteria and *Bifidobacterium lactis* BB-12® were above 8 log CFU/g. Higher viability of all bacteria was obtained in yoghurt fortified with 1.5 % quince powder than those of other samples after 14 days. Probiotic yoghurt with added 1.5 % quince powder had the highest proteolytic activity during the last two weeks of storage, while the highest total phenolic content and (2,2-diphenyl-1-picrylhydrazyl) radical (DPPH˙) scavenging activity were obtained for the same sample throughout the storage period.

**Novelty and scientific contribution:**

Quince powder supports the health of the digestive system thanks to its high fibre content, while it is also rich in vitamins, minerals and antioxidant activity. These properties emphasise the importance of using quince powder in yoghurt production. This innovation has the potential to provide consumers with a tasty alternative, while at the same time increasing the intake of health-promoting ingredients. Furthermore, such products offer higher nutritional value than conventional yoghurt, offering consumers with a healthier option. Therefore, the production of yoghurt with quince powder can be considered as an important innovation in the field of nutrition and a practice that contributes to increasing health awareness.

## INTRODUCTION

According to Food and Agriculture Organization of the United Nations (*1*) food production in the world should increase due to the increasing population. As more than 30 % of the produced food is wasted, this waste adversely affects food security and also comes at a high environmental cost. Therefore, in recent years, valorisation of fruit and vegetable by-products has received a great attention to support waste management. The use of quince-based products has been a subject of various studies, especially due to its high antioxidant activity. Bayav and Şahin (*2*) state that the market for processed quince-based products should grow to increase quince consumption and exports.

Quince (*Cydonia oblonga*) is a member of *Maloideae* subfamily that belongs to *Rosaceae* family. It shows a high variability according to geographical origin, maturity, climate, cultivar and genetics. Due to its hardness and astringent taste, it is mostly used to produce marmalade, jam, puddings and jelly (*3*). Quince is a good source of pectin, dietary fibre and minerals and also has a high antioxidant activity that depends on its phenolic content (*4*). The phenolic content and antioxidant activity of quince have been found to be higher than those of some other fruits such as apple and pear (*5*, *6*). In addition, quince-based products have been reported to have beneficial effects on many diseases such as diarrhoea, ulcer, dysentery, constipation, diabetes, atherosclerosis and respiratory disorders (*7*).

Functional foods are considered as foods that, in addition to basic nutrition, offer health benefits and reduce the risk of chronic diseases (*8*). Functional dairy products, such as probiotic yoghurt, are already known as healthy due to the beneficial effect of viable bacteria. The use of fruit-based additives in yoghurt formulation has gained considerable interest in recent years to improve the product functionality. Probiotic yoghurt supplemented with a natural source of antioxidants provides consumers with nutrients, healthy effects of starter bacteria and antioxidant compounds, mainly phenolic compounds. An improvement in the viability of culture bacteria and/or antioxidant activity of grape extract (*9*), apple and banana fibre (*10*), passion fruit peel powder (*11*), jambolan fruit pulp (*12*) and pineapple peel (*13*) in yoghurts have been reported by many authors.

In recent studies, quince fruit has been used in different forms in the production of some dairy products and the properties of the final product have been investigated. For example, the positive effects of adding quince seed mucilage powder, quince boiling water or quince seed gel to different types of yoghurt and drinking yoghurt, such as doogh, have been shown in some studies (*14*-*16*). The enhancing effect of quince seed gum on the viability of *Bifidobacterium lactis* BB-12® in drinking yoghurt, doogh, was observed on the first day of storage compared to the control sample (*17*). On the other hand, the effects of quince seed, quince powder and quince seed gum on some other dairy products such as ice cream and whipped cream have also been studied (*18*, *19*). In another study, quince seed mucilage was used as a coating material in a dairy dessert containing microencapsulated *Lacticaseibacillus rhamnosus* and the obtained results indicated good maintenance of probiotic viability in microencapsulation with quince seed mucilage (*20*). However, there is a lack of information about the use of whole quince powder in the production of probiotic yoghurt and the effect of this addition on the viability of both yoghurt starter bacteria and *B. lactis* and also proteolytic and antioxidant activities of yoghurt.

The aim of this study is to investigate the use of whole quince powder in the production of probiotic yoghurt and its effect on product quality throughout 28 days of storage. In this context, the influence of the addition of different amounts of quince powder on viable counts of bacteria, the total phenolic content, radical scavenging activity and proteolytic activity of probiotic yoghurt containing *B. lactis* BB-12 were determined.

## MATERIALS AND METHODS

### Materials

Fresh quinces of Gordes variety were purchased from a market in Izmir, Turkey. The freeze-dried direct vat set (DVS) yoghurt culture (*Streptococcus thermophilus* and *Lactobacillus delbrueckii* ssp. *bulgaricus* and *Bifidobacterium animalis* ssp. *lactis* BB-12® were obtained from Chr. Hansen A/S, Hørsholm, Denmark, and contained 10^11^ and 10^10^ CFU/g, respectively. Nonfat milk powder was obtained from Pınar Dairy Products, Pınarbası, Izmir, Turkey.

### Preparation of quince powder

After washing, the quinces were peeled, cored and sliced into 1-mm thick slices before drying and grinding. Quince peels and slices were dried and powdered separately under the same conditions. The mixture of 20 % quince peel powder and 80 % quince slice powder were used in the production of yoghurt.

The drying was carried out in a tray dryer (Armfield Ltd., Ringwood, Hampshire, UK). One mm thick quince slices were spread in a thin layer on the tray (dimensions 17.5 cm×22.5 cm). The inlet and outlet temperatures were (80±2) and (78±2) °C, respectively. The air velocity of the dryer was set at 1 m/s. The quince slices that came out of the dryer were ground with a blender (KB 6234 T B-Fit®; Arçelik, Istanbul, Turkey), hermetically packaged and stored under dry and dark conditions until the quince powder was used. Bags made of aluminium polyethylene (ALPE) were used for packaging the powders. The details of processing and powder properties were given in the study by Çınar *et al.* (*21*).

### Yoghurt production

Whole milk with 4 % milk fat was used for the production of the experimental probiotic yoghurts. The solid content of nonfat milk was adjusted to 120 g/L with nonfat milk powder and then the standardised milk was divided into four samples. Three samples were enriched with 0.5, 1.0 or 1.5 % quince powder, while the fourth sample, without any addition, served as a control. After pasteurisation at 85 °C for 30 min, the preparations were cooled to 43 °C. Each milk base was inoculated with both yoghurt starter culture (*L. bulgaricus* and *S. thermophilus*) and *B. lactis* BB-12. Yoghurt starter culture was prepared by adding the freeze-dried commercial starter to 1 L of sterilised milk at 40 °C under aseptic conditions. After that, the prepared yoghurt starter culture (*L. bulgaricus* and *S. thermophilus*) was added to milk according to the manufacturer’s instructions. Then, the freeze-dried *B. lactis* BB-12 (0.02 %) culture was added to obtain a probiotic viability of 8 log CFU/g in the product. The mixtures were then fermented at 42 °C until pH=4.65. After fermentation, probiotic yoghurts were cooled at room temperature for about 30 min and refrigerated at 4 °C until use.

### pH value

A pH meter (model 211; Hanna Instruments, Woonsocket, RI, USA) was used for the determination of pH values. The pH values of the yoghurt samples were measured weekly for 28 days.

### Microbiological analyses

After preparing appropriate decimal dilutions, viable counts were determined according to Akalın *et al.* (*22*). The viable counts of *S. thermophilus* were determined using M17 agar (Merck, Darmstadt, Germany) after an aerobic incubation at 37 °C for 72 h, while the viability of *L. bulgaricus* was detected in de Man, Rogosa and Sharpe (MRS) agar after an anaerobic incubation at 42 °C for 72 h. Viable cells of *B. lactis* BB-12 were enumerated on MRS-NNLP (nalidixic acid, neomycin sulfate, lithium chloride and paramomycin sulfate) agar. The plates were incubated anaerobically at 37 °C for 72 h using anaerobic jars (Merck).

### Proteolytic activity

The proteolytic activity was measured using the *o*-phtaldialdehyde (OPA) method, which is based on measuring the absorbance of a solution of amino acids and peptides (*23*). In the method, OPA reagent (50 mL of 1000 mmol/L sodium tetraborate, 5 mL of 20 % sodium dodecyl sulphate, 80 mg of OPA dissolved in 2 mL of methanol and 200 μL of β-mercaptoethanol topped up with dH_2_O to a final volume of 100 mL) was used and the absorbance of the solutions was measured at 340 nm using a spectrophotometer (Spekol 1300; Analytik Jena AG, Jena, Germany). The water extract of yoghurt was prepared by mixing 5 g of yoghurt sample with 5 mL of 24 % trichloroacetic acid (Sigma-Aldrich, Merck) and the mixture was left at room temperature for 1 h. After centrifugation (centrifuge model Sigma 3-16K; Sigma Laborzentrifugen GmbH, Osterode am Harz, Germany) of the mixture at 3743×*g* and 4 °C for 20 min, it was filtered through Whatman no. 42 filter paper. The permeate was added to 3 mL of OPA reagent and the absorbance of the solution was measured spectrophotometrically at 340 nm after 2 min at room temperature (20 °C). The proteolytic activity was expressed as the absorbance value.

### Total phenolic content and antioxidant activity

The Folin-Ciocalteu method was used to determine the total phenolic content of experimental yoghurts (*24*, *25*). Yoghurt extracts were prepared according to Singleton *et al*. (*25*). For this purpose, 10 g of yoghurt sample were centrifuged (model Sigma 3-16K; Sigma Laborzentrifugen GmbH) at 9383×*g* for 25 min, filtered through Whatman no. 1 filter paper and the supernatant was used in the method. A volume of 0.1 mL of sample extract was mixed with 6 mL of dH_2_O and 0.5 mL Folin-Ciocalteu reactive reagent (Carlo Erba Reagents, Val de Reuil, France) and left for 2 min. Then, 1.5 mL of 20 % sodium carbonate (Merck, Darmstadt, Germany) were added. After keeping the mixture at room temperature in the dark for 2 h, the absorbance was read at 760 nm using a spectrophotometer (Spekol 1300; Analytik Jena AG). A gallic acid standard curve was calculated using the following equation with the correlation coefficient R^2^=0.9987:


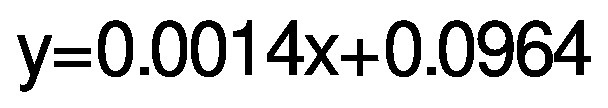
 /1/

and the results were expressed in milligrams of gallic acid equivalents (GAE) per litre of sample.

The 2,2,-diphenyl-1-picrylhydrazyl (DPPH˙) scavenging activity of the samples was determined according to Unal and Akalın (*26*). A 0.1 mmol/L DPPH˙ solution in 95 % ethanol was prepared. A volume of 8 mL of ethanolic DPPH˙ solution was placed in a 50-mL centrifuge tube and mixed with 2 mL of sample or 95 % ethanol (as control), vortexed for 5 s and then incubated for 30 min at room temperature. The samples were then centrifuged (centrifuge model Sigma 3-16K; Sigma Laborzentrifugen GmbH) for 5 min at 9383×*g* and room temperature. Supernatants were filtered through Whatman no. 40 filter paper. The absorbance of each sample was measured at 517 nm. 6-Hydroxy-2,5,7,8-tetramethylchroman-2-carboxylic acid (Trolox) was used as a reference antioxidant at a concentration of 0.25 mg/mL. DPPH˙ scavenging activity was calculated according to the following equation:


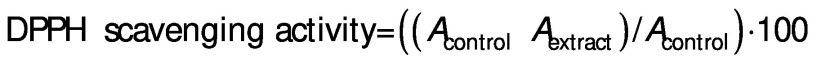
 /2/

### Statistical analysis

The experiment was repeated three times and the analyses were carried out at least in duplicate, *i.e.* six samples from each yoghurt type were evaluated in each analysis. The statistical analysis was carried out by one-way ANOVA using the general linear model (SPSS Statistica v. 25) (*27*). The significant differences were determined by Duncan’s multiple comparison test at the p<0.05 level.

## RESULTS AND DISCUSSION

The physical properties of the quince powder were determined in our previous study (*21*). The moisture content, dispersibility and water-holding capacity values of quince powders were determined to be 3.07 %, 65.36 % and 3.05 g/g, respectively. The composition (total solids, fat and protein) of the experimental yoghurts was also analysed in our previous study (*21*). There was no significant difference in the fat (between (3.73±0.06) and (3.9±0.6) %) and protein (between (4.3±0.2) and (4.4±0.0) %) mass fraction among yoghurt samples, while total solids content of yoghurts increased significantly with the increase in quince powder amount (p<0.05).

### Changes in pH values

**Fig. 1:**
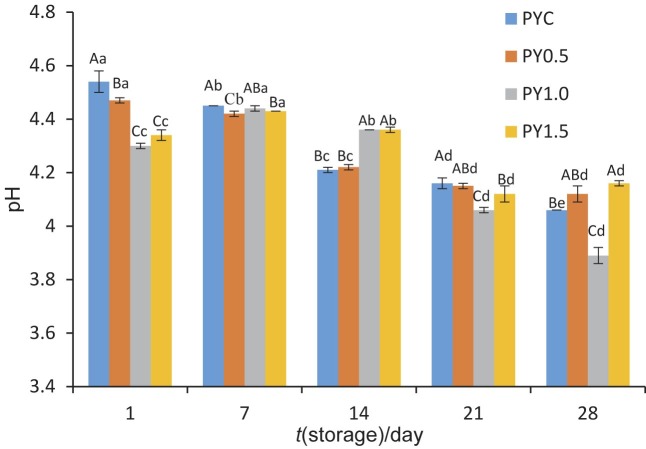
Changes in pH values during refrigerated storage of yoghurts (error bars represent SD). PYC=probiotic yoghurt control without supplement, PY0.5, 1.0 and 1.5=probiotic yoghurt fortified with 0.5, 1.0 and 1.5 % quince powder, respectively. Different uppercase letters indicate significant differences among samples for the same storage period (p<0.05). Different lowercase letters indicate significant differences among different storage periods of the same sample (p<0.05)

Fig. 1 shows the pH changes of probiotic yoghurts during 28 days of refrigerated storage. A reduction was observed in the control sample and the yoghurt enriched with 0.5 % quince powder throughout the storage period. The pH values of the yoghurts supplemented with 1 and 1.5 % quince powder increased on day 7, but then decreased during the rest of the storage (p<0.05). The pH values were significantly influenced by the addition of quince powder. The probiotic yoghurts with added quince powder had generally lower pH values than the control sample. The lowering effect of quince powder on pH can be due to the quince composition and its beneficial effect on viable counts of starter culture bacteria in our yoghurt samples (Table 1). The lowest pH values were obtained for yoghurt supplemented with 1 % quince powder on the 1^st^, 21^st^ and 28^th^ day of storage.

**Table 1:** Viable counts of *Streptococcus thermophilus*, *Lactobacillus delbrueckii* ssp. *bulgaricus* and *B. lactis* during refrigerated storage of yoghurts

	*t*(storage)/day				
Product	1	7	14	21	28
	*N*(viable cell)/(log CFU/g)				
	*S. thermophilus*				
PYC	(9.06±0.01)^Ba^	(8.81±0.00)^ABa^	(8.2±0.2)^Bb^	(8.8±0.3)^Ba^	(8.9±0.2)^Ba^
PY0.5	(8.8±0.2)^Bab^	(8.4±0.4)^Bb^	(7.92±0.06)^Bc^	(9.1±0.3)^ABa^	(9.09±0.03)^Aa^
PY1.0	(8.8±0.2)^Ba^	(9.07±0.03)^Aa^	(8.2±0.4)^Bb^	(8.8±0.1)^Ba^	(9.08±0.08)^Aa^
PY1.5	(9.4±0.2)^Aa^	(8.9±0.2)^Abc^	(8.97±0.06)^Abc^	(9.2±0.2)^Aab^	(8.9±0.1)^Bc^
	*L. delbrueckii* ssp. *bulgaricus*				
PYC	(9.20±0.04)^Ba^	(9.39±0.04)^Aa^	(8.4±0.4)^Bc^	(8.70±0.03)^Cb^	(8.49±0.04)^Dbc^
PY0.5	(9.21±0.08)^Ba^	(8.3±0.4)^Bb^	(8.2±0.1)^Bb^	(9.38±0.03)^Aa^	(9.39±0.06)^Aa^
PY1.0	(8.59±0.08)^Cb^	(9.2±0.3)^Aa^	(8.10±0.05)^Bc^	(8.79±0.04)^Bb^	(9.07±0.02)^Ba^
PY1.5	(9.6±0.1)^Aa^	(9.1±0.1)^Ac^	(9.25±0.02)^Abc^	(9.34±0.06)^Ab^	(8.6±0.1)^Cd^
	*B. lactis*				
PYC	(9.07±0.04)^Ba^	(8.87±0.05)^Ab^	(8.60±0.06)^Bc^	(8.54±0.03)^Bc^	(8.5±0.2)^Bc^
PY0.5	(9.06±0.03)^Bb^	(8.22±0.03)^Bd^	(8.04±0.07)^Ce^	(9.20±0.05)^Aa^	(8.83±0.06)^Ac^
PY1.0	(9.3±0.1)^Aa^	(8.85±0.01)^Ab^	(8.0±0.1)^Cd^	(8.67±0.08)^Bc^	(8.78±0.04)^Abc^
PY1.5	(9.3±0.1)^Aa^	(8.84±0.01)^Abc^	(9.0±0.1)^Ab^	(8.68±0.07)^Bc^	(8.4±0.2)^Bd^

PYC=probiotic yoghurt control without supplement, PY0.5, 1.0 and 1.5=probiotic yoghurt fortified with 0.5, 1.0 and 1.5 % quince powder, respectively. Mean values±standard deviation in the same row with different lowercase letters in superscript are significantly different (p<0.05). Mean values±standard deviation in the same column with different uppercase letters in superscript are significantly different (p<0.05)

The pH values were also higher in the control yoghurt than in the sample with added 0.2 % quince seed mucilage powder, which was obtained by preparing aqueous quince seed solution, then applying agitation, filtration, evaporation and lyophilisation processes. The authors associated this with a correlation between the pH value and *L. bulgaricus* viability (*16*). Similarly, Trigueros *et al.* (*14*) measured lower pH values of low-fat yoghurt enriched with quince boiling water than of control yoghurt during fermentation. Lower pH values were determined for fibre-supplemented yoghurts than for the control group in other studies (*10*, *28*). On the other hand, no significant effect of adding fruit fibre on the pH values of yoghurts was observed in a previous research (*9*, *29*, *30*). This difference from our study can be related to the different sources and forms of fruit fibre used in the production of yoghurt.

### Microbiological properties

The viability of *Streptococcus thermophilus*, *Lactobacillus bulgaricus* and *B. lactis* during storage is shown in Table 1. The viable cell counts of yoghurt starter bacteria and *B. lactis* were above 8 log CFU/g during 28 days.

Quince powder addition had a promoting effect on the viability of *B. lactis* in general. Higher viable counts were enumerated in the sample containing 1.5 and 0.5 % quince powder during the first two weeks and the last two weeks of storage, respectively. Although the viability of *B. lactis* significantly decreased on the 28^th^ day compared to the 1^st^ day, it remained two log units above the recommended minimum value (10^6^ CFU/g) (*31*) in all samples. The highest viability of *B. lactis* was found in the samples with added 1 and 1.5 % quince powder (PY1.0 and PY1.5, respectively) in the first two weeks of storage and in the sample with added 0.5 % quince powder (PY0.5) in the last two weeks. It has been reported that different fruit fibre increased the viability of *Bifidobacterium* species in fermented milk and yoghurt matrix (*10*, *11*, *32*, *33*).

Pectin, which is a soluble dietary fibre found in various plant foods including quince, has a prebiotic effect and inhibits intestinal pathogens (*34*). Moreover, Thomas *et al.* (*35*) found that pectins are mostly found in the quince flesh, while the skin is quite rich in non-fibre compounds such as proteins and lipids. Therefore, the stimulating effect of quince powder on the viability of *B. lactis* can be due to the fact that most of the quince powder content used in our study (80 % quince slice powder and 20 % quince peel powder) consists of the fleshy part of the quince. In addition, Miletić *et al.* (*34*) reported that quince pectin-derived oligosaccharides act as a prebiotic and increase the amount of bifidobacteria probably due to various substances such as flavan-3-ol (epicatechin), procyanidin B2, 8-hydroxycinnamates, caffeoylquinic (CQA) and coumaroylquinic acid derivatives, kaempherol and quercetin derivatives, as well as monosaccharides.

The probiotic yoghurt fortified with 0.5 % quince powder had the highest viable counts of *B. lactis* on days 21 and 28 probably due to higher pH values on those days than other samples. The pH value of 4.5 and lower negatively affects the viability of *Bifidobacterium* species in yoghurt at 5 °C (*36*). Viable counts of *L. bulgaricus* were also found highest in PY0.5 on days 21 and 28 in our study. This is in agreement with the results obtained by Espírito Santo *et al.* (*10*), who found an increase in the viable cell counts of both *L. bulgaricus* and *B. lactis* HN019 in yoghurt towards the end of the 28 days. The authors reported that a symbiotic relationship between the fruit fibte and *B. lactis* HN019 may result in increased viability of *L. bulgaricus* in yoghurt. On the other hand, the simultaneous increase in the viability of both yoghurt culture bacteria and *B. lactis* in our study, which was also seen in the PY1.5 sample in the first two weeks, could also be a result of the symbiotic relationship between them. It has been reported that free amino acids formed by *L. bulgaricus* increase the growth of *S. thermophilus* and *Bifidobacteria* (*37*).

The addition of quince powder significantly affected the survival of *S. thermophilus* and the highest viability during storage was determined in the probiotic yoghurt with 1.5 % quince powder. On the other hand, there was no statistically significant difference between the yoghurts with 0.5 or 1.0 % quince powder. Although a decrease (p<0.05) in the viable counts of *S. thermophilus* was observed in yoghurts PYC, PY0.5 and PY1.0 on day 14, the values on day 28 did not change statistically compared to the beginning of storage. However, a fluctuation in the viability of *S. thermophilus* was observed during the storage of yoghurt supplemented with 1.5 % quince powder and a significant decrease in the number of viable bacteria was observed on day 28 compared to the beginning of storage.

The addition of quince powder to probiotic yoghurt generally improved the survival of *L. bulgaricus*. Gürbüz *et al.* (*16*) reported that the fortification with quince seed mucilage powder had a beneficial effect on the viability of *L. bulgaricus*, but the effect was not significant on *S. thermophilus* in set-style yoghurt. Trigueros *et al.* (*14*) found significantly higher counts of *S. thermophilus* and *L. bulgaricus* (p<0.05) in control yoghurt than in the samples with added quince boiling water during 28 days. The authors reported that this was correlated with the lower pH value of control yoghurts.

In some other studies (*10*, *11*, *13*, *38*), an improving effect of the addition of fruit fibre on the viability of *S. thermophilus* and *L. bulgaricus* in probiotic yoghurts was observed. An increased growth of yoghurt bacteria was also obtained by adding the extract of blackcurrant polyphenols to yoghurt formulation before fermentation (*39*). Furthermore, the survival of yoghurt bacteria was also improved in fermented milk containing *B. bifidum* CECT 870 with orange or apple fibre (*32*). Chouchouli *et al.* (*29*) did not report any significant effect of grape seed extract on the viable counts of *Lactobacillus* populations throughout the shelf life of four weeks. The differences between the studies may be due to the different pectin polymerisation degrees of the dietary fibre in the used fruit. Bazzocco *et al*. (*40*) reported the relationship between the degree of polymerisation of a polysaccharide and its viability due to its fermentability.

### Proteolytic activity of probiotic yoghurt

Table 2 shows that proteolytic activity of the probiotic yoghurt samples varied between *A*_340 nm_=0.63 and 0.95 and fluctuated during storage. The proteolytic activity of all yoghurt samples significantly increased (p<0.05) on days 7 and 14 and then there was no significant change until the end of storage. The increase in proteolytic activity was significant and dose-dependent in the yoghurts with quince powder compared to the control yoghurt without any additions. The proteolytic activity is estimated by the degree of protein hydrolysis carried out by the proteolytic enzymes of the culture bacteria. The peptide bonds of milk proteins are cleaved, which results in the formation of peptides and amino acids (*41*). Therefore, it is obvious that the positive effect of quince powder on the viability of culture bacteria is a consequence of the proteolytic activity of the supplemented yoghurts.

**Table 2:** Proteolysis values of probiotic yoghurts

	*t*(storage)/day				
Product	1	7	14	21	28
	*A*_340 nm_				
PYC	(0.63±0.00)^Dc^	(0.67±0.02)^Db^	(0.74±0.01)^Da^	(0.73±0.01)^Da^	(0.73±0.00)^Ca^
PY0.5	(0.67±0.00)^Cd^	(0.73±0.00)^Cc^	(0.81±0.00)^Cb^	(0.83±0.00)^Cab^	(0.85±0.06)^Ba^
PY1.0	(0.72±0.01)^Bc^	(0.77±0.00)^Bb^	(0.84±0.00)^Ba^	(0.84±0.01)^Ba^	(0.84±0.01)^Ba^
PY1.5	(0.73±0.01)^Ac^	(0.89±0.01)^Ab^	(0.89±0.00)^Ab^	(0.95±0.00)^Aa^	(0.95±0.01)^Aa^

PYC=probiotic yoghurt control without supplement, PY0.5, 1.0 and 1.5=probiotic yoghurt fortified with 0.5, 1.0 and 1.5 % quince powder, respectively. Mean values±standard deviation in the same row with different lowercase letters in superscript are significantly different (p<0.05). Mean values±standard deviation in the same column with different uppercase letters in superscript are significantly different (p<0.05)

Sah *et al*. (*13*) investigated the effect of the addition of pineapple peel powder on some properties of probiotic yoghurt. Yoghurt supplemented with pineallpe peel powder had higher proteolytic and stronger antioxidant activity than plain yoghurt. The study attributed the high proteolysis to the proteolytic activity of the starter culture. The authors also determined a positive but weaker correlation between the degree of proteolysis and DPPH˙ scavenging capacity (r=0.76) compared to other antioxidant capacity methods [reducing power (r=0.91), ABTS (r=0.89) and hydroxyl radicals (r=0.98)]. In our study, we also found a positive but weak correlation (r=0.576) between DPPH˙ scavenging activity and proteolytic activity.

### Total phenolic content and antioxidant activity

**Fig. 2:**
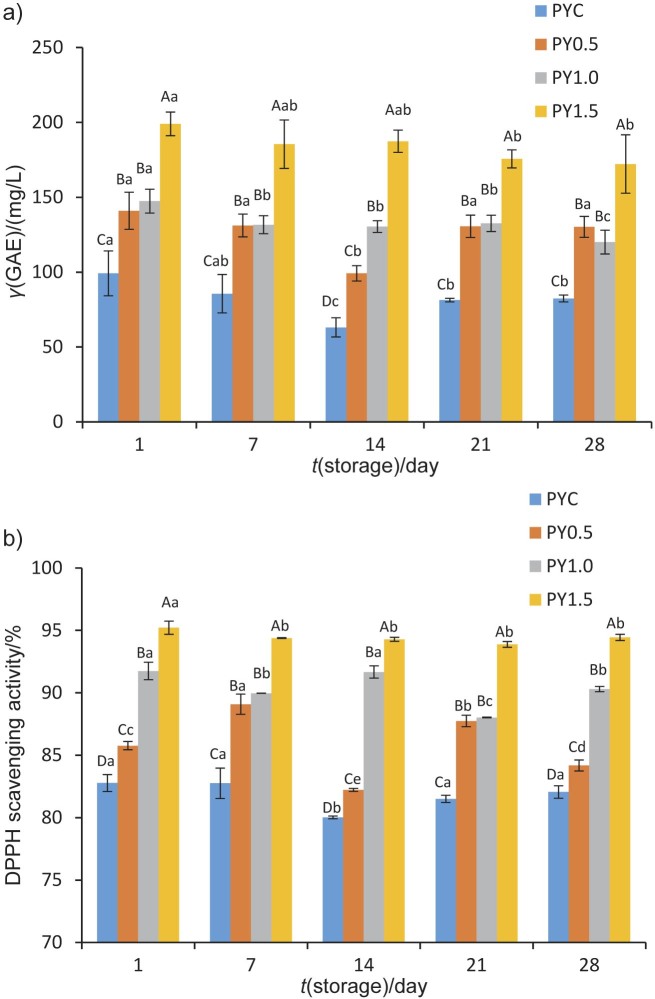
The results of: a) total phenolic content expressed as gallic acid equivalents (GAE) and b) antioxidant activity during storage (error bars represent SD). PYC=probiotic yoghurt control without supplement, PY0.5, 1.0 and 1.5=probiotic yoghurt fortified with 0.5, 1.0 and 1.5 % quince powder, respectively. Different uppercase letters indicate significant differences among samples during the same storage period (p<0.05). Different lowercase letters indicate significant differences within the same sample among different storage periods (p<0.05)

The total phenolic content (TPC) and radical scavenging activity of probiotic yoghurts are shown in Fig. 2a and Fig. 2b, respectively. The TPC, espressed as gallic acid equivalents (GAE), of yoghurt samples was between 63.11 and 199.00 mg/L. The DPPH˙ scavenging activity of the yoghurts was between 80.02 and 95.22 %. The reference antioxidant, Trolox, at a concentration of 0.25 mg/mL, showed a radical scavenging activity of 97.09 %.

Fortification of yoghurt with quince powder significantly increased the TPC (p<0.05). Probiotic yoghurt enriched with 1.5 % quince powder had the highest TPC, while the control sample had the lowest values throughout storage. On the other hand, there were no significant differences in TPC (p>0.05) between the samples with added 0.5 and 1.0 % quince powder. The higher TPC in the PY1.5 sample may be mainly due to the quince powder content and higher viability of *L. bulgaricus* among probiotic yoghurts. Tabasco *et al.* (*42*) reported that *L. bulgaricus* can metabolise phenolic compounds, which results in increased phenolic content. Although there was a fluctuation in the TPC values of our experimental yoghurts during storage, a decrease (p<0.05) was detected at the end of 28 days compared to the 1^st^ day, probably due to the degradation of phenolic compounds.

The addition of quince powder significantly affected the antioxidant activity of probiotic yoghurt and this effect varied with amount increase. Sample PY1.5 had the highest antioxidant activity for 28 days (p<0.05). As it has previously been reported, phenolic compounds have an important role in scavenging free radicals and providing hydrogen to prevent oxidation reactions (*43*). Therefore, as the antioxidant activity is strongly correlated with the quantity of phenolic compounds, the result obtained in our study is expected. A significant and robust positive correlation between total phenolic content and DPPH˙ scavenging activity of quince was also found by Silva *et al*. (*44*).

The high radical scavenging activity of sample PY1.5 could also be due to a high viability of the culture, which leads to the release of bioactive peptides with antioxidant properties during fermentation. In fact, sample PY1.5 also had higher viable counts (Table 1) and proteolytic activity (Table 2). Farvin *et al.* (*45*) investigated the antioxidant capacity of yoghurt fractions and concluded that the bioactive peptides with antioxidant properties could act as electron donors and thus create stable molecules from free radicals. The scavenging activities against DPPH˙ observed in all experimental yoghurt samples generally did not change significantly during storage.

Quince is known to be rich in potential antioxidants such as phenolic acids and flavonoids, and it has been suggested that caffeic acid and its derivatives are mainly responsible for the antioxidant capacity of quince (*46*). Najman *et al.* (*47*) determined caffeic acid mass fraction of 3.50 mg/100 g in quince powder obtained by convective drying at 70 °C, similar to our drying method, while the value for fresh quince was 0.61 mg/100 g. Studies have shown that higher phenolic content and antioxidant activity were found in quince peel than in the flesh part and whole fruit (*3*, *6*, *48*–*51*). Sonmez and Sahin (*51*) also found a higher TPC and DPPH˙ scavenging activity in quince peel extract than in seed extract. Therefore, the presence of peel in the quince powder in this study provides an advantage in terms of antioxidant properties.

Grygorieva *et al.* (*48*) investigated the properties of phenolic compounds, free radical scavenging activity (DPPH˙ method) and molybdenum-reducing antioxidant power of different genotypes of quince fruit. The authors showed that all parameters differed significantly among the studied genotypes. They found significant positive correlations between the DPPH˙ scavenging activity and both total polyphenols and phenolic acids in the samples of Ukrainian origin. It was also reported that the pectin content, composition and physicochemical properties of quince vary according to the genotype. Qin *et al*. (*52*) investigated different pectic fractions of cell wall polysaccharides from quince. They determined that the DPPH˙ scavenging activity of all the studied fractions was affected by the pectin concentration. Furthermore, chelator-soluble pectin had the highest DPPH˙ scavenging activity (85 %) among the pectic fractions. Wojdyło *et al.* (*53*) suggested that there are different pathways for polyphenols to scavenge free radicals. They reported that the low-molecular-mass antioxidants (chlorogenic acid, (−)-epicatechin) present in quince are involved in the first step of the reaction with the radical. The polymerised procyanidins then react with free radicals after 10 min or later and thus develop antioxidant activity.

It has been shown that the total phenolic content and antioxidant activity of quince and foods with added quince can vary depending on the presence of certain polyphenolic compounds that are crucial for free radical scavenging activity (*50*). Moreover, various factors can influence these parameters, such as the genotype and geographical origin of the quince, its maturity, powder composition, especially the peel content and the antioxidant method used (*54*, *55*).

## CONCLUSIONS

In this study, a high viability of the yoghurt starter bacteria and *Bifidobacterium lactis* BB-12® was achieved in all probiotic yoghurts throughout storage. The addition of quince powder to the yoghurt improved the viability of *B. lactis* and also the yoghurt starter bacteria and increased the antioxidant and proteolytic activities. The highest viability of *Streptococcus thermophilus*, *Lactobacillus bulgaricus* and *B. lactis* was observed in the yoghurt with 1.5 % quince powder for 14 days, while the yoghurt with 0.5 % quince powder had the highest probiotic viable counts during the remaining storage days. Probiotic yoghurt fortified with 1.5 % quince powder had the highest antioxidant properties and proteolytic activity during refrigerated storage. The use of quince powder in the production of yoghurt, especially at amount of 1.5 %, offers the advantage of obtaining a dairy product with improved functional properties. This study showed the beneficial use of fruit waste, so the results are a guideline for further research in both functional food science and waste management.
